# Development and testing of a tailored online fertility preservation decision aid for female cancer patients

**DOI:** 10.1002/cam4.3711

**Published:** 2021-02-13

**Authors:** Michelle van den Berg, Elleke van der Meij, Annelies M. E. Bos, Marieke C. S. Boshuizen, Domino Determann, Ramon R. J. P. van Eekeren, Christianne A. R. Lok, Eva E. Schaake, Petronella O. Witteveen, Marielle J. Wondergem, Didi D. M. Braat, Catharina C. M. Beerendonk, Rosella P. M. G. Hermens

**Affiliations:** ^1^ Department of Obstetrics and Gynecology Radboud University Medical Center Nijmegen The Netherlands; ^2^ Department of Obstetrics and Gynecology University Medical Center Utrecht Utrecht The Netherlands; ^3^ PATIENT+ Utrecht The Netherlands; ^4^ Department of Surgery Rijnstate Hospital Arnhem The Netherlands; ^5^ Centre for Gynecological Oncology Amsterdam The Netherlands Cancer Institute‐Antoni van Leeuwenhoek Hospital Amsterdam The Netherlands; ^6^ Department of Radiotherapy The Netherlands Cancer Institute‐Antoni van Leeuwenhoek Hospital Amsterdam The Netherlands; ^7^ Department of Medical Oncology University Medical Center Utrecht Utrecht The Netherlands; ^8^ Department of Hematology VU University Medical Center Amsterdam Amsterdam The Netherlands; ^9^ Department of IQ Healthcare Radboud University Medical Center Nijmegen The Netherlands

**Keywords:** cancer, decision aid, fertility preservation, shared decision making, tailored information

## Abstract

**Background:**

Decision making regarding future fertility can be very difficult for female cancer patients. To support patients in decision making, fertility preservation decision aids (DAs) are being developed. However, to make a well‐informed decision, patients need personalized information tailored to their cancer type and treatment. Tailored cancer‐specific DAs are not available yet.

**Methods:**

Our DA was systematically developed by a multidisciplinary steering group (n = 21) in an iterative process of draft development, three rounds of alpha testing, and revisions. The drafts were based on current guidelines, literature, and patients' and professionals' needs.

**Results:**

In total, 24 cancer‐specific DAs were developed. In alpha testing, cancer survivors and professionals considered the DA very helpful in decision making, and scored an 8.5 (scale 1–10). In particular, the cancer‐specific information and the tool for recognizing personal values were of great value. Revisions were made to increase readability, personalization, usability, and be more careful in giving any false hope.

**Conclusions:**

A fertility preservation DA containing cancer‐specific information is important in the daily care of female cancer patients and should be broadly available. Our final Dutch version is highly appraised, valid, and usable in decision making. After evaluating its effectiveness with newly diagnosed patients, the DA can be translated and adjusted according to (inter)national guidelines.

## Graphical abstract

A fertility preservation decision aid tailored to cancer type and associated cancer treatments was systematically developed for female cancer patients. The final version is highly appraised, valid, and usable in decision‐making and should be broadly available.

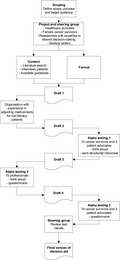

## 1 INTRODUCTION

Improved survival rates for cancer patients of reproductive age have increased the importance of addressing long‐term side effects of cancer treatments.^1^ Potential loss of fertility due to the gonadotoxicity of cancer treatments is an important long‐term side effect for female cancer survivors of reproductive age.^2^ Therefore, guidelines recommend that the risk of infertility and fertility preservation (FP) options should be discussed before the start of the cancer treatment.^3^, ^4^ Current FP options include cryopreservation of oocytes, embryos, and ovarian tissue, ovarian transposition, ovarian suppression, and fertility sparing surgery.

Patients want to be informed about the effects of cancer treatment on their fertility and the available FP options via written and/or digital information in order to make a well‐informed decision.^5^, ^6^ However, studies have shown that in current care not all patients are informed on these risks and options, and patients have reported unmet information needs.^7^, ^8^, ^9^ Even if information on fertility risks and options is provided, decision making regarding future fertility is very difficult and complex. The decision has to be made in a very short time frame in a period with great emotional distress in which patients and their partners focus on surviving cancer and not on their future fertility.^6^ In addition, not all FP options are appropriate for all patients. Dependent on patient's age, relationship status, cancer type, cancer treatment, prognosis, and the amount of time before starting cancer treatment, some preservation options are more appropriate than others. As a consequence, patients experience decisional conflict regarding this decision. Decisional conflict increases if patients did not obtain enough information on all FP options, and if patients did not feel supported during decision making.^9^, ^10^, ^11^ This suboptimal care in information provision and support increases concerns regarding fertility and long‐term regret, affecting female cancer patients' quality of life negatively.^2^, ^12^, ^13^

Therefore, it is important that female cancer patients are well informed and supported in their decision regarding FP. Providing a decision aid (DA) is a way to support patients in this complex decision‐making process. DAs are described as evidence‐based tools designed to support patients in making choices among healthcare options. They provide evidence‐based information on the options, associated benefits, and harms, and help patients to recognize their personal values in the decision‐making process. DAs increase patients' knowledge and decrease their decisional conflict compared to usual care.^14^

A recent study reviewed and evaluated nine FP DAs.^15^ These DAs significantly increased FP knowledge and decreased decisional conflict. Furthermore, they were found to be helpful, contained relevant information, and patients reported high levels of satisfaction with their use. Only three of these nine DAs are currently available for female cancer patients: one for breast cancer patients, and two not specific to any cancer type, in Portuguese and in German.^16^, ^17^, ^18^

However, DAs that personalize information based on cancer type and treatment are not available yet. Therefore, the aim of this study was to develop and test an online Dutch FP DA tailored to cancer type and associated cancer treatments and infertility risks for female cancer patients of reproductive age.

## 2 METHODS

### 2.1 Development process

**FIGURE 1:**
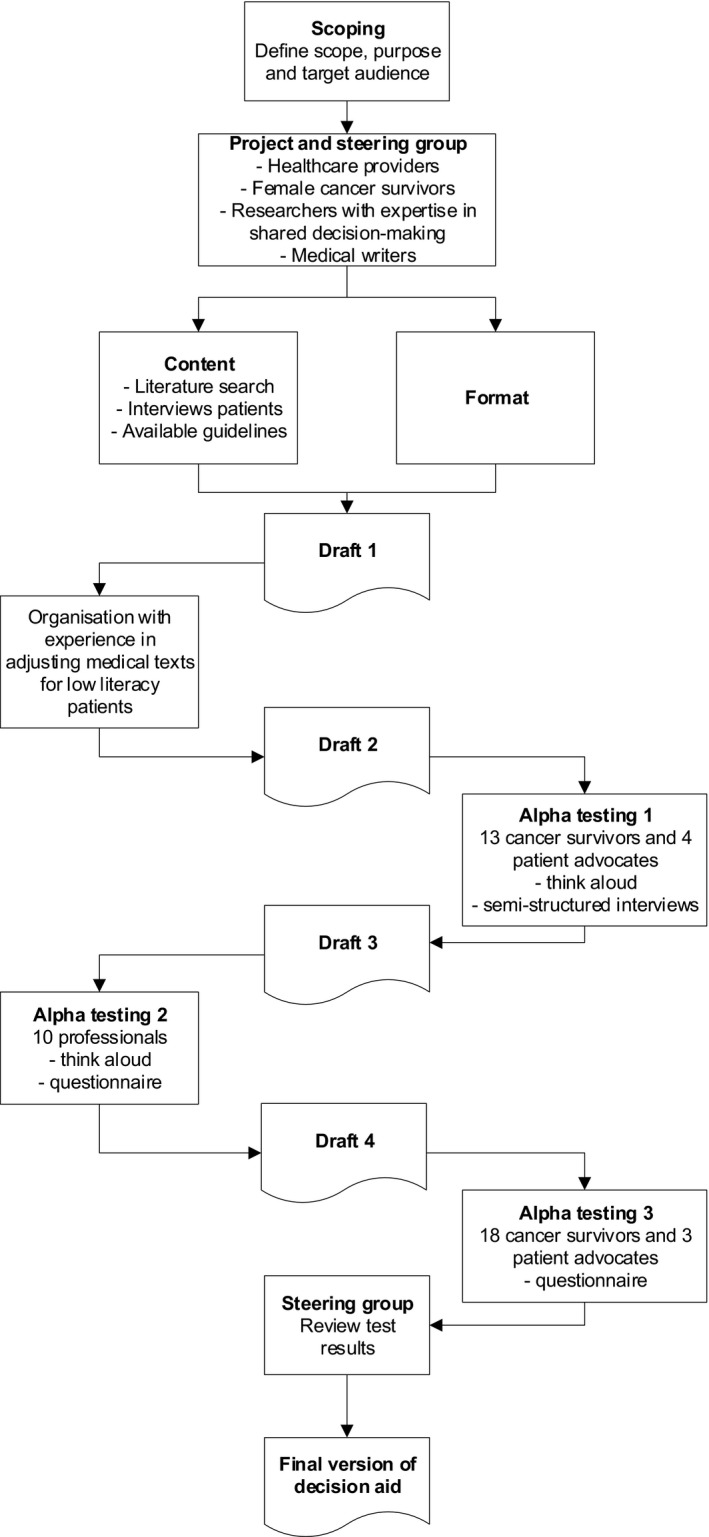
Systematic development process of the fertility preservation decision aid

The FP DA was systematically developed in 2019/2020 using the recommendations published by Coulter et al.^19^ and in accordance with the international patient DA standards (IPDAS) criteria.^20^ The development process is shown in Figure 1 and was performed by a project group (N = 6) guided by a multidisciplinary steering group (N = 15) consisting of healthcare professionals working in female oncofertility care throughout the Netherlands, female cancer survivors and patient advocates (affiliated with a patient association). They were recruited from the working group who developed the Dutch FP guideline in 2016.^4^ This study was approved by the Medical Ethical Committee of the Radboudumc (2018–4996), and all participants provided written consent to participate.

#### 2.1.1 Setting

In the Netherlands, cancer patients receive multidisciplinary oncological care and can be referred for specialized FP care by any medical specialist involved. At three Dutch hospitals, all FP options, including ovarian tissue cryopreservation, are performed. All legal residents of the Netherlands are obliged by law to have basic health insurance, which covers FP counseling and all FP options, meaning that patients have no financial reasons to refrain from it.

#### 2.1.2 Scope and purpose

The scope and purpose of the DA was defined by the project group. It should be part of the implementation of the national FP guideline. A meeting was held with the project and steering group to reach consensus on the scope, purpose, target audience, and moment of providing the DA.

#### 2.1.3 Content and format

##### Patients' needs and preferences—data collection

To explore patients' needs and preferences in a FP DA, in‐depth interviews were conducted with female cancer survivors who had FP counseling and made a decision on FP treatment in the past. They were recruited from an academic medical center. A topic list guided the interviews and survivors were asked for their opinion about: the content, the format, ways to personalize the DA, a value clarification exercise, how much time they want to spent on the DA, and the moment of providing the DA. The interviews were conducted by M.B. (researcher); took place at the academic medical center or by telephone, depending on patients' preferences; and lasted approximately 30 min. The number of interviews was determined by data saturation (the point at which no new information was mentioned).

##### Patients' needs and preferences—data analysis

All interviews were audio recorded, transcribed verbatim, and analyzed through grounded theory analysis using qualitative research software Atlas.ti (version 8.2, Berlin).^21^ Data were anonymized, and the transcripts were not returned to participants for comments or feedback. The coding process consisted of the following steps. Each step was performed independently by two researchers to increase reliability and validity. All interviews were read first. Second, both researchers selected and labeled phrases describing experiences or improvement suggestions, using open encoding (ie, using patients' own words). The descriptive codes that showed resemblance were combined and redefined into specific subthemes. These subthemes were then merged into broader themes by using axial coding. The broader themes formed the conceptual model for patients' needs and preferences that was devised by using the grounded theory method. After each step, the results were compared, and any discrepancies were discussed until consensus was reached. In addition, each interview was analyzed directly, so new topics could be added to the initial topic list.

##### Professionals' needs and preferences—data collection

As part of our previous study, 24 oncological healthcare providers and reproductive specialists were interviewed about their barriers and improvement suggestions in female oncofertility care.^6^ They were recruited from the three Dutch expertise hospitals for female FP care (Radboudumc, LUMC, and ErasmusMC) and their affiliated hospitals (ca.20). All interviews were conducted by M.B., and depending on professional's preference, interviews were conducted in person or via telephone, and lasted approximately 40 min. An interview guide was used to standardize the interview process, and the number of interviews was determined by data saturation.

##### Professionals' needs and preferences—data analysis

Analysis of professionals' needs and preferences was part of our previous study and is described in our published paper.^6^ In short, coding of the interviews was independently performed by two authors and was guided by Flottorp's framework.^22^

##### Content

Besides the interviews, a literature review was performed to provide information on current infertility risks and pregnancy chances of FP options. The following domains were important in FP decision making according to professionals' interviews and the literature review^6^: (1) infertility risks associated with cancer treatment; (2) burden and risks of FP treatment; (3) pregnancy chances associated with FP options; (4) consequences of the decision for future fertility; (5) patients' personal values in decision making. The content of these domains was tailored according to patients' needs and preferences. The current national FP guideline formed the basis for the content of domain 1–4.^4^ For the fifth domain, important items to clarify patients' values in decision making were extracted from patients' interviews.

##### Format

An online, Web‐based, format was chosen by the project and steering group to be able to provide a DA that is tailored to a patient's cancer type.

### 2.2 Methods of alpha testing and revision

#### 2.2.1 Data collection

After an iterative process of reviewing and revising the content with the project and steering group, the first draft was evaluated by an organization with experience in adjusting medical texts for low literacy patients (www.stichtingmakkelijklezen.nl). The second draft was evaluated with female cancer survivors who made a decision regarding FP in the past, and with patient advocates. They were recruited from an academic medical center and patient associations. All interviews were conducted by M.B., and the number of interviews was determined by data saturation. The first part of the interview was unstructured according to the think aloud method.^23^ The second part was semi‐structured, and participants were asked about the content, layout, comprehensibility, usability, and acceptability of the DA.^24^, ^25^, ^26^ Based on the received feedback, a third draft was developed and alpha‐tested with professionals working in oncofertility care, as suggested by Coulter,^19^ recruited from the steering group. They were also invited for an interview, conducted by M.B., using the think aloud method,^23^ and were asked to fill in a questionnaire with questions about the content, clearness, and usefulness, and were asked to rate the DA.^25^, ^26^ The number of interviews was, again, determined by data saturation. Finally, a revised fourth draft was quantitatively evaluated with female cancer survivors and patient advocates using a questionnaire similar to the professionals' questionnaire. They were, again, recruited from an academic medical center and patient associations. In addition to the quantitative evaluation, the quality of the fourth draft was tested against the 64 IPDAS criteria.

#### 2.2.2 Data analysis

Again, all patients' and professionals' interviews were audio recorded, transcribed verbatim, and analyzed using qualitative research software Atlas.ti (version 8.2, Berlin). Patients' and professionals' data were anonymized and analyzed separately. The transcripts were not returned to participants for comments or feedback. The coding process consisted of the following steps and was performed by two authors (M.B. and E.M.). All interviews were read first. Second, both authors selected and labeled phrases describing feedback and improvement suggestions per chapter of the decision aid, using open encoding (ie, using patients' own words). All feedback and improvement suggestions were listed and discussed in a face‐to‐face meeting with the project group. After reaching consensus with the project group on which suggestions should be incorporated in the new draft, all decision aids were revised and evaluated in the next round of alpha testing. Patients' and professionals' questionnaires were analyzed using SPSS (version 25.0 for Windows). Regarding the IPDAS criteria, all members of the project group evaluated the 64 IPDAS criteria independently, where after consensus was reached in a face‐to‐face meeting.

## 3 RESULTS

### 3.1 Development process

#### 3.1.1 Scope and purpose

The scope and purpose of the DA was twofold. The main scope was to support female cancer patients in their decision whether they want to undergo a FP treatment or not. Furthermore, the DA also supports patients in the decision which FP treatment they want to undergo. The steering group agreed that the DA should be provided before FP counseling with a reproductive gynecologist and is meant to be complementary to the counseling. Furthermore, the target audience was defined as female cancer patients of reproductive age (>18 years) who have to undergo a gonadotoxic cancer treatment. No cancer types were excluded.

#### 3.1.2 Content and format

##### Patients' needs and preferences—results

Nine out of nineteen female cancer survivors participated in the in‐depth interview about their needs and preferences in a FP DA. Their mean age was 32 years (SD 6,8 years), they were diagnosed with breast cancer (78%) or Hodgkin's lymphoma (22%), and all had undergone a FP treatment.

Regarding the content, all patients mentioned that the DA should be developed per cancer type, so they do not have to read information that is not applicable to them. Furthermore, the most important topics to provide were the consequences of cancer treatment for fertility and the live birth rate per FP treatment. Patients expressed a need to know their personal risk of infertility in order to make a decision, meaning that the risk of infertility should be provided per cancer treatment. The burden, risks, pros, and cons of each FP option should be extensively provided. In addition, some patients wanted to read about egg donation, adoption, foster care, and surrogacy and the steps to go through if you have a wish to conceive after surviving cancer, because patient information about these topics is lacking at this moment in the Netherlands.

Another topic in the interviews was the way the DA should be personalized. As stated before, all patients wanted a DA personalized to their cancer type. Regarding FP treatments, patients wanted to read about the following options: “wait and see,” oocyte, embryo, and ovarian tissue cryopreservation, even if one of those options was not applicable to them. They did not want to have the feeling that options were deliberately withheld from them. However, it should be very clear for whom the treatment is applicable.

All patients wanted to use the DA online and wanted to spent about 30 min on the DA. Most patients would use the DA before counseling with a reproductive gynecologist; however, some patients mentioned they had been so overwhelmed by the cancer diagnosis that they would use the DA after counseling.

##### Professionals' needs and preferences—results

In total, 24 out of 43 individual professionals agreed to participate in the interviews about their barriers and improvement suggestions in female oncofertility care. Regarding barriers in information provision, professionals mentioned that there was a lack of written and digital information for patients. Furthermore, professionals mentioned that patients had a need for FP information tailored to their personal situation to be able to make a decision.

##### Content and format

**FIGURE 2:**
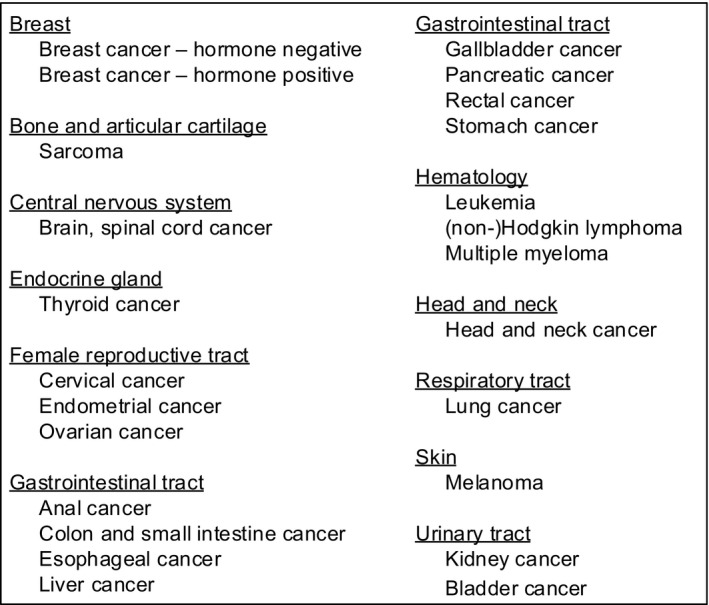
Overview of the 24 developed cancer‐specific decision aids

Based on patients' and professionals' needs and preferences, the project and steering group decided to develop 24 cancer‐specific DAs, one for each cancer type occurring in young females (Figure 2). Patients have access to one specific DA, that is, access to the DA specific to their cancer type. All DAs were divided into five chapters: Information; Comparison of options; Important items; Your preference and values; and Closure, and arranged in the same way and are comparable to each other. There is one exception, the DAs for gynecological cancers are really different from all other DAs, as the cancer treatment itself could already be a FP option. The first chapter “Information” begins with an introduction for whom the DA is applicable and the purpose. Thereafter, general information about cancer treatment and infertility is provided. In the next section, for each cancer type (ie, different in each DA) tailored information about all possible cancer treatments that could be given for that specific cancer type is presented including their consequences for fertility and uterine function, if applicable. After reading about infertility risks, the decision is displayed, “wait and see” versus “FP treatment” including all applicable treatments for that specific cancer type. In the next pages, extensive information about the following topics is provided for all options; for whom the option is appropriate, explanation about treatment, live birth rate, safety, risks, pros, and cons. For example, for breast cancer patients, oocyte and embryo cryopreservation are presented as good and applicable options, and for leukemia patients, these are presented as not so applicable, as there might be no time before starting cancer treatment. Furthermore, an image has been developed to explain each treatment. This chapter ends with information about the steps to go through when a patient has a wish to conceive after surviving cancer, including alternative family building options. In chapter 2, patients can compare all FP options including “wait and see” in an interactive table. To personalize this table, patients can check and uncheck all options and all above‐mentioned topics. In chapter 3, patients are asked to answer basic questions about cancer and fertility to check whether they understood the information. In the fourth chapter, patients are asked to fill in a value clarification exercise containing nine statements to recognize their personal values in decision making. In the final chapter, patients are asked to fill in three questions to clarify if they had gained enough knowledge, learned about their values in this decision, and if they were prepared for the FP counseling consultation.

### 3.2 Results of alpha testing and revision

To increase readability, wording, sentences and tables were changed in the first draft. For example, “FP options” was changed into “possibilities to have children in the future,” enumerations were used to clarify complex sentences, and the text was shortened and detailed information was removed from tables to increase readability for patients with a low health literacy.

#### 3.2.1 Alpha testing round 1

Consensus was reached on the adjustments with the steering group, and the second draft was ready for evaluation. In total, 17 out of 24 female cancer survivors and patient advocates consented to participate. Their characteristics are shown in Table 1. Overall, they were satisfied with the content and layout and considered it very helpful in decision making. In particular, the cancer‐specific information and the tool for recognizing personal values were of great value. The information was comprehensible, and the images were very illustrative. All cancer survivors would have liked to use the DA if this would have been available. However, they also had suggestions to improve the DA. Regarding the content, they suggested to add information about the process of accepting that a patient might never have children, to move information about alternative family building options from “wait and see” to “wish to conceive after surviving cancer,” and to be careful in giving any false hope or wrong expectations. To increase usability, it was suggested to clarify navigation through the DA. Cancer survivors also suggested to personalize the DA more by choosing which FP options to read about instead of going through all options. Last, to increase readability, they suggested to make icon arrays and tables visible at a glance and to add colors. This led to major changes in the third draft.

**TABLE 1:** Characteristics of female cancer survivors and patient advocates in alpha testing round 1 and round 3

	Alpha testing round 1		Alpha testing round 3	
	Cancer survivors (N = 13)	Patient advocates (N = 4)	Cancer survivors (N = 18)	Patient advocates (N = 3)
Mean age in years (range)				
Current	35.3 (23–49)	52.7 (43–69)	34.8 (23–49)	56 (43–70)
At diagnosis	28.6 (19–35)		28.4 (19–35)	
Gender (% female)	100%	75%	100%	67%
Education level (N, %)				
Secondary education	1/13 (7.7%)			
Vocational education	2/13 (15.4%)	1/4 (25%)	3/18 (16.7%)	1/3 (33%)
Higher professional education	5/13 (38.5%)	2/4 (50%)	6/18 (33.3%)	1/3 (33%)
University degree	5/13 (38.5%)	1/4 (25%)	9/18 (50%)	1/3 (33%)
Diagnosis (N, %)				
Breast cancer	5/13 (38.5%)		7/18 (38.9%)	
Cervical cancer	4/13 (30.8%)		5/18 (27.8%)	
Endometrial cancer			1/18 (5.6%)	
Hodgkin's disease	2/13 (15.4%)		2/18 (11.1%)	
Ovarian cancer	1/13 (7.7%)		1/18 (5.6%)	
Soft tissue sarcoma	1/13 (7.7%)		1/18 (5.6%)	
Vulvar cancer			1/18 (5.6%)	
Advocate of (N, %)				
Breast cancer		2/4 (50%)		2/3 (67%)
Hematological malignancy		1/4 (25%)		1/3 (33%)
Gynecological malignancy		1/4 (25%)		
Mean years of experience as advocate (range)		7.0 (3–9)		7.7 (5–10)
Cancer treatment (N, %)				
Surgery breast	5/13 (38.5%)		7/18 (38.9%)	
Surgery reproductive organs	4/13 (30.8%)		7/18 (38.9%)	
Chemotherapy	11/13 (84.6%)		14/18 (77.8%)	
Radiotherapy (reproductive organs)	3/13 (23.1%)		3/18 (16.7%)	
Targeted therapy	1/13 (7.7%)		1/18 (5.6%)	
Endocrine therapy	2/13 (15.4%)		2/18 (11.1%)	
Relationship status at diagnosis (N, %)				
Single	4/13 (30.8%)		5/18 (27.8%)	
In a relationship	5/13 (38.5%)		8/18 (44.4%)	
Married	4/13 (30.8%)		5/18 (27.8%)	
Parity at diagnosis				
Nulliparous	10/13 (76.9%)		15/18 (83.3%)	
Parous	3/13 (23.1%)		3/18 (16.7%)	
Strength of wish to conceive on a scale of 1–10 (mean, range)			7.1 (2–10)	
Fertility discussed (N, %)				
Yes	11/13 (84.6%)		15/18 (83.3%)	
No	2/13 (15.4%)		3/18 (16.7%)	
Fertility preservation counseling received (N, %)				
Yes, by reproductive gynecologist	7/13 (53.8%)		10/18 (55.6%)	
Yes, by gynecological oncologist	4/13 (30.8%)		2/18 (11.1%)	
No	2/13 (15.4%)		6/18 (33.3%)	
Fertility preservation treatment (N, %)				
Yes	10/13 (76.9%)		10/18 (55.6%)	
Oocyte cryopreservation	3/10		3/10	
Embryo cryopreservation	1/10		1/10	
Ovarian tissue cryopreservation	2/10		3/10	
Ovarian transposition	3/10		3/10	
Fertility sparing surgery	3/10		2/10	
Hormonal ovarian suppression	1/10		1/10	
Combined treatments	3/10		3/10	
No	3/13 (23.1%)		8/18 (44.4%)	
Pregnancy pursued (N, %)	1/13 (7.7%)		3/18 (16.7%)	
Stored material used (N, %)	0		1/18 (5.6%)	

#### 3.2.2 Alpha testing round 2

The third draft was evaluated with 10 professionals. Their characteristics are shown in Table S1. In general, professionals were very satisfied with the content, format, and layout of the DA. They had fewer suggestions to improve the DA than survivors had. They suggested to add a disclaimer to emphasize that the information is based on current guidelines, but is subject to change over time. In addition, they suggested to clarify which treatment (cancer or FP) is meant in various places throughout the DA. Specifically for the gynecological DAs, it was suggested to alter the images of the fertility sparing treatments to make them more clear. Results from the questionnaire are shown in Table 2. All professionals would recommend the use of the DA and scored it with an 8.5 (scale 1–10, SD 0,53). All suggestions were included in the fourth draft.

**TABLE 2:** Alpha testing among female cancer survivors, patient advocates, and healthcare professionals

	Draft 3 Healthcare professionals (n = 10)	Draft 4 Female cancer survivors and patient advocates (n = 21)
Time spent in minutes (range)	30 (20–50)	24 (10–60)
Length		
Too long	0	0
Too short	0	0
Just right	10 (100%)	21 (100%)
Amount of information		
Too much	0	1 (4.8%)
Too little	0	1 (4.8%)
Just right	9 (90%)	19 (90.4%)
Missing	1 (10%)	
Information balanced?		
Yes	9 (90%)	21 (100%)
No, leaning toward wait and see	0	0
No, leaning toward fertility preservation	0	0
Missing	1 (10%)	
DA comprehensible in general?		
Very good	2 (20%)	11 (52.4%)
Good	8 (80%)	10 (47.6%)
Moderate	0	0
Bad	0	0
Risks comprehensible?		
Very good	5 (50%)	6 (28.5%)
Good	4 (40%)	14 (66.7%)
Moderate	0	1 (4.8%)
Bad	0	0
Missing	1 (10%)	
DA clear?		
Very good	4 (40%)	9 (42.9%)
Good	6 (60%)	12 (57.1%)
Moderate	0	0
Bad	0	0
Information appropriate for patients?		
Very good	8 (80%)	10 (47.6%)
Good	2 (20%)	8 (38.1%)
Moderate	0	2 (9.5%)
Bad	0	0
Missing		1 (4.8%)
Navigation through DA?		
Very good	6 (60%)	10 (47.6%)
Good	3 (30%)	9 (42.9%)
Moderate	1 (10%)	1 (4.8%)
Bad	0	0
Missing		1 (4.8%)
Credibility?		
Very	3 (30%)	13 (61.9%)
Moderate	7 (70%)	8 (38.1%)
A little	0	0
Not at all	0	0
Confusing items?		
Yes	4 (40%)	6 (28.5%)
No	6 (60%)	15 (71.5%)
Images helpful?		
Very	8 (80%)	15 (71.5%)
Moderate	1 (10%)	6 (28.5%)
A little	0	0
Not at all	0	0
Missing	1 (10%)	
Personal value clarification helpful?		
Made choice easier	7 (70%)	11 (52.4%)
Made choice harder	0	0
Does not influence choice	2 (20%)	9 (42.9%)
Missing	1 (10%)	1 (4.8%)
DA helpful in decision making?		
Very	9 (90%)	12 (57.1%)
Moderate	1 (10%)	8 (38.1%)
A little	0	1 (4.8%)
Not at all	0	0
Average score on scale of 1–10 (range)?	8.5 (8–9)	8.5 (7–10)
Recommend use of DA? (%)	100%	100%

#### 3.2.3 Alpha testing round 3

The fourth draft was sent to 23 female cancer survivors and patient advocates of whom 21 responded (Table 1). Results from the questionnaire are shown in Table 2. The DA was scored as balanced, clear, comprehensible, and 95% would find it useful in decision making. Confusing items were reported by 29%, this concerned word use for which suggestions were made, the usability of the comparison table, and clearness of risks. All participants would recommend the DA to others and scored the DA with an 8,5 (scale 1–10, SD 0,75). Based on the improvement suggestions, a final version of the DAs was drafted with the project and steering group.

#### 3.2.4 IPDAS criteria

The checklist of the IPDAS collaboration was used to assess the quality of our DA.^20^ A total of 45 out of 64 criteria on the checklist were applicable to our study. Criteria on the field‐testing and effectiveness were not applicable as this has not been evaluated yet. The final version of the DA met 43 out of the 45 (96%) applicable IPDAS criteria (Table S2). In the content domain, all 23 criteria were met. Regarding the development process domain, 19 out of 21 criteria were met. We did not meet the criteria that the online DA allows patients to search for key words. Furthermore, the criteria that patients received feedback on personal entered information were not met, as patients did not have to enter personal information because the DA was already cancer‐specific.

## 4 DISCUSSION

This paper described the systematic development process of 24 cancer‐specific FP DAs for female cancer patients by a multidisciplinary steering group. All DAs addressed risks, safety, pros, and cons of “wait and see,” and of all applicable FP treatments. The final versions were considered clear, appropriate, usable, and helpful in decision making by female cancer survivors, patient advocates, and their healthcare professionals. Furthermore, 43 out of 45 quality criteria for content and development process of the IPDAS checklist were met.

This is the first study in which a FP DA tailored to all cancer types and treatment was developed. In previous studies, FP DAs have been developed either for one specific cancer type or not specific to any cancer type.^15^ These DAs proved to be effective in improving knowledge and reducing decisional conflict. The structure of these DAs is the same as ours; however, tailoring information to a patient's individual situation has also shown to be very important in making high‐quality decisions. In the study of Ehrbar et al, tailored information for three specific cancer types (breast cancer, lymphoma, and ovarian cancer) was provided, but not for all cancer types.^27^ In a narrative review, a wide range of factors was found to impact the FP decision‐making process, including a patient's personal situation, and a patient's dilemma of being in the survival mode or to prioritize FP treatment.^28^ Another qualitative study explored breast cancer patients' experiences with FP discussions and information.^29^ Patients reported a strong desire to have their individual preferences and personal situations addressed during fertility discussions, and therefore, predetermined FP information would not be appropriate. These studies emphasize the need of tailoring information to patient's specific values and preferences, which can be done by providing our tailored DA.

Noteworthy, our study underlines the importance of involving patients in all stages during the development of the DA. Most studies describing the development of a FP DA also involved patients in the development process, however not throughout all stages.^27^, ^30^, ^31^, ^32^ For example in the study of Jones et al., patients were not involved in the first stage, developing the first draft.^30^ Our DA would have been different if patients were not involved throughout all stages. To develop the first draft of our DA, female cancer survivors were members of our steering group, and we explored patients' needs and preferences in decision making. This led to the development of DAs that were cancer‐specific, and provided information about a patient's personal risk of infertility. If we did not involve them in the first stage of development, we would not have developed 24 cancer‐specific DAs. Furthermore, our statements in the value clarification exercise were based on patients' values extracted from the interviews. In other studies, it was not clear whether the statements were based on patients' values.^27^, ^30^, ^31^, ^32^ Our exercise was based on the whole process of decision making, that is, whether to undergo a FP treatment or not, which is different to other DAs.^27^, ^31^, ^32^ We chose to do it like this, because of two reasons. First, this was the main scope of our DA. Second, interviewed patients mentioned that this exercise would help them in making the decision whether to undergo a FP treatment or not, but not in which FP treatment to choose. They wanted to discuss that in more detail with their reproductive gynecologist. In the next stages of development, other female cancer survivors were asked to evaluate the DAs where after major changes were made to increase the usability and readability of the DA. So, although patients were involved in developing the first draft, the final versions of the DAs were still considerably different underlining the importance to involve patients throughout all steps of DA development.

A strength of our study is that it is one of the few studies in which three consecutive rounds of alpha testing and revisions were conducted to optimize the DA. Our number of participants (female cancer survivors N = 38, professionals N = 10) in alpha testing was higher than in most studies (female cancer survivors N = 10–20, professionals N = 7–17).^27^, ^30^, ^31^, ^32^, ^33^, ^34^ This ensured we involved female cancer survivors with all types of cancers and treatments, of all reproductive ages, and of whom some had undergone a FP treatment, while others had not. Our study showed that this was of utmost importance in the development of the gynecological cancer DAs, as major changes were made according to feedback during alpha testing.

Some limitations, despite the systematic development according to international standards, should be considered in the interpretation of the results. Although we included a high number of female cancer survivors in alpha testing, the DAs were not tested with newly diagnosed cancer patients who might have different information and decision support needs. However, an advantage of testing with female cancer survivors is that they were also aware of the consequences of their decision. This provided us with additional information that newly diagnosed patients probably could not have overseen. Furthermore, bias could have occurred because most female cancer survivors had a partner and a strong wish to conceive before decision making. Patients who were single and who had doubts about their wish to conceive may make their decision based on different information and values. In addition, most female cancer survivors were highly educated which may bias the results regarding the comprehensibility and usability of the DA. However, to minimize this bias we involved an organization with experience in adjusting medical texts for low literacy patients. Another limitation of our study was that we did not involve partners. Partners may play an important role in FP decision making; however, most patients indicated that it was their own decision and their partner did not play a crucial role. Furthermore, we only involved heterosexual Caucasian patients, who may make decisions based on other cultural values than sexual or racial minorities. Last, although the information in the DAs was tailored and cancer‐specific, it should only be used complementary to FP counseling and should not replace counseling. Oncological healthcare providers should still refer all patients to a reproductive specialist to discuss the information in the DA.

The next step in our development process is to field‐test the DAs in “real life” conditions with patients and professionals not involved in the development process.^19^ This will lead to the final version of the DA, which is then ready for implementation into daily clinical practice. This implementation will be facilitated, because multiple key stakeholders, both healthcare providers working in oncology care and reproductive medicine, and patient associations, were already involved in the development process.^35^ Thereafter, it should be evaluated if the DA reduces decisional conflict and decision regret regarding FP decision making. Our final version of the FP DA can be translated and adjusted according to local and (inter)national guidelines and available FP options to make it broadly available for female cancer patients.

In conclusion, a FP DA tailored to cancer type and associated cancer treatments was systematically developed for female cancer patients of reproductive age. The DA aims to support patients in well‐informed FP decision making based on their personal situations and preferences. The involvement of healthcare providers, female cancer survivors, and patient associations led to a final version of the DA that is highly appraised, valid, and usable in decision making. After field‐testing and evaluating the impact on decision making in newly diagnosed patients, the DA will be available in the Netherlands, and internationally, after translation and adjustment to international guidelines and locally available FP options.

## CONFLICT OF INTEREST

The authors report no conflict of interest.

## Supporting information

Table S1

Table S2

## Data Availability

Study data are available upon request.
