# Supplementary material for: Development and testing of a tailored online fertility preservation decision aid for female cancer patients
Source: Cancer Med. 2021 Feb 13;10(5):1576–88. doi: 10.1002/cam4.3711 (PMC7940215; doi:10.1002/cam4.3711)
Supplement: Supplementary file 1 — Table S1 [file CAM4-10-1576-s001.docx]

**Supplementary Table 1. Professionals’ characteristics in alpha testing round 2**

|  | Professionals (N=10) |
| --- | --- |
| Mean age in years(range) | 47,1(36-65) |
| Gender (%Female) | 90% |
| Type of professional(N,%)  Gynecological oncologist  Hematologist  Medical oncologist  Oncological surgeon  Radiation oncologist  Reproductive gynecologist  Specialized oncology nurse  Specialized fertility nurse | 1(10%)  1(10%)  1(10%)  1(10%)  1(10%)  2(20%)  1(10%)  2(20%) |
| Type of hospital(N,%)  Academic hospital  Non-academic hospital  Cancer center | 7(70%)  1(10%)  2(20%) |
| Years of experience as professional(N,%)  0-5 years  5-10 years  10-15 years  15-20 years  > 20 years | 1(10%)  3(30%)  3(30%)  0  3(30%) |
| Number of oncofertility patients professional treats yearly(N,%)  0-10  10-20  20-30  30-40  >40 | 0  2(20%)  4(40%)  2(20%)  2(20%) |
