# Supplementary material for: Development and testing of a tailored online fertility preservation decision aid for female cancer patients
Source: Cancer Med. 2021 Feb 13;10(5):1576–88. doi: 10.1002/cam4.3711 (PMC7940215; doi:10.1002/cam4.3711)
Supplement: Supplementary file 2 — Table S2 [file CAM4-10-1576-s002.docx]

**Supplementary Table 2. International Patient Decision Aid Standards (IPDAS) Checklist**

| **Criteria** | **Answer** |
| --- | --- |
| **Domain 1. Content: Does the patient decision aid…** |  |
| ***Provide information about options in sufficient detail for decision-making?*** |  |
| 1.Describe the health condition | Yes |
| 2.List the options | Yes |
| 3.List the option of doing nothing | Yes |
| 4.Describe the natural course without options | Yes |
| 5.Describe procedures | Yes |
| 6.Describe positive features (benefits) | Yes |
| 7.Describe negative features of options (harms / side effects / disadvantages) | Yes |
| 8.Include changes of positive / negative outcomes | Yes |
| ***Present probabilities of outcomes in an unbiased and understandable way?*** |  |
| 9.Use event rates specifying the population and time period | Yes |
| 10.Compare outcome probabilities using the same denominator, time period, scale | Yes |
| 11.Describe uncertainty around probabilities | Yes |
| 12.Use visual diagrams | Yes |
| 13.Use multiple methods to view probabilities (words, numbers, diagrams) | Yes |
| 14.Allows the patient to select a way of viewing probabilities (words, numbers, diagrams) | Yes |
| 15.Allow patient to view probabilities based on their own situation (e.g. age) | Yes |
| 16.Place probabilities in context of other events | Yes |
| 17.Use both positive and negative frames (e.g. showing both survival and death rates) | Yes |
| ***Include methods for clarifying and expressing patients’ values?*** |  |
| 18.Describe the procedures and outcomes to help patients imagine what it is like to experience their physical, emotional, social effects | Yes |
| 19.Ask patients to consider which positive and negative features matter most | Yes |
| 20.Suggest ways for patients to share what matters most with others | Yes |
| ***Include structured guidance in deliberation and communication?*** |  |
| 21.Provide steps to make a decision | Yes |
| 22.Suggest ways to talk about the decision with a health professional | Yes |
| 23.Include tools (worksheet, question list) to discuss options with others | Yes |
| **Domain 2. Development process: does the patient decision aid…** |  |
| ***Present information in a balances manner?*** |  |
| 24.Able to compare positive / negative features of options | Yes |
| 25.Shows negative / positive features with equal detail (fonts, order, display of statistics) | Yes |
| ***Have a systematic development process*** |  |
| 26.Include developers’ credentials / qualifications | Yes |
| 27.Finds out what users (patients, practitioners) need to discuss options | Yes |
| 28.Has peer review by patient / professional experts not involved in development and field testing | Yes |
| 29.Is field tested with users (patients facing the decision; practitioners presenting options) | Not yet |
| The field test with users (patients, practitioners) show the patient decision aid is:  30.Acceptable  31.Balanced for undecided patients  32.Understood by those with limited reading skills | N/A  N/A  N/A |
| ***Use up to date scientific evidence that is cited in a reference section or technical document?*** |  |
| 33.Provides references to evidence used | Yes |
| 34.Reports steps to find, appraise, summarize evidence | Yes |
| 35.Report date of last update | Yes |
| 36.Report how often patient decision aid is updated | Yes |
| 37.Describe quality of scientific evidence (including lack of evidence) | Yes |
| 38.Uses evidence from studies of patients similar to those of target audience | Yes |
| ***Disclose conflicts of interest?*** |  |
| 39.Report source of funding to develop and distribute the patient decision aid | Yes |
| 40.Report whether authors or their affiliations stand to gain or lose by choices patients make after using the patient decision aid | Yes |
| ***Use plain language?*** |  |
| 41.Is written at a level that can be understood by the majority of patients in the target group | Yes |
| 42.Is written at a grade 8 equivalent level or less according to readability score (SMOG or FRY) | Yes |
| 43.Provides ways to help patients understand information other than reading (audio, video, in-person discussion) | Yes |
| ***Meet additional criteria if the patient decision aid is internet based*** |  |
| 44.Provide a step-by-step way to move through the web pages | Yes |
| 45.Allow patients to search for key words | No |
| 46.Provide feedback on personal health information that is entered into the patient decision aid | No |
| 47.Provides security for personal health information entered into the decision aid | Yes |
| 48.Make it easy for patients to return to the decision aid after linking to other web pages | Yes |
| 49.Permit printing as a single document | Yes |
| **Domain 3. Effectiveness: does the patient decision aid ensure decision making is informed and values based?** |  |
| ***Decision processes leading to decision quality. The patient decision aid helps patients …*** |  |
| 50.Recognize a decision needs to be made | N/A |
| 51.Know options and their features | N/A |
| 52.Understand values that affect decision | N/A |
| 53.Be clear about option features that matter most | N/A |
| 54.Discuss values with their practitioner | N/A |
| 55.Become involved in preferred ways | N/A |
| ***Decision quality. The patient decision aid…*** |  |
| 56.Improves the match between the chosen option and the features that matter most to the informed patient | N/A |
